# Excitonic Enhancement and Excited Excitonic States in CsPbBr_3_ Halide Perovskite Single Crystals

**DOI:** 10.3390/ma16010185

**Published:** 2022-12-25

**Authors:** Anna Yu. Samsonova, Vsevolod I. Yudin, Anna V. Shurukhina, Yury V. Kapitonov

**Affiliations:** Department of Photonics, Saint-Petersburg State University, ul. Ulyanovskaya 1, 198504 Saint-Petersburg, Russia

**Keywords:** halide perovskites, exciton, reflectivity

## Abstract

Halide perovskites are novel photonics materials promising numerous applications in fields such as photovoltaics, LED light sources, microlasers, and radiation detectors. Many halide perovskites are direct-gap semiconductors, and Wannier–Mott excitons play a significant role in their optical properties near the fundamental absorption edge. The high oscillator strength of these states favors applications where efficient interaction with light is required. In this work, to study excitonic states in CsPbBr${}_{3}$ halide perovskite single crystals, the reflection spectroscopy at temperatures from 4 K was used. A reflection coefficient up to 70% was observed for the n=1 exciton state, followed by weak excited states of excitons with n=2 and n=3. It should be noted that the Sommerfeld enhancement factor should be considered for a correct description of the behavior of the dielectric constant, taking into account excitonic effects.

## 1. Introduction

Halide perovskite semiconductors have attracted considerable research interest due to the cheap synthesis and many possible applications in fields of optics and optoelectronic devices, such as light-emitting diodes Ref. [1], lasers, Ref. [2], gamma- and X-ray detectors [3] and photodetectors [4]. The ABX${}_{3}$ (A${}^{+}$ = Cs${}^{+}$, CH${}_{3}$NH${}_{3}^{+}$, X${}^{-}$ = I${}^{-}$, Br${}^{-}$ or Cl${}^{-}$) perovskites are known to be direct band-gap hybrid organic–inorganic halide semiconductors with an organic or inorganic A-cation located in the hollows formed by a framework of corner-sharing BX${}_{6}$ inorganic octahedra. The stability of such an infinite structure is provided by a suitable size of the A cation, predicted by the Goldschmidt tolerance factor [5] for ionic solids. At relatively low temperatures, the optical properties near the band gap are determined by exciton resonances. In 3D halide perovskites with an exciton binding energy of the order of tens of meV, depending on the exact material, these excitonic effects play a major role at temperatures close to or even exceeding room temperature. It was shown that hydrogen-like Wannier–Mott excitons are observed in these materials [6,7]. Accurate experimental characterization of excitons was carried out for several more 3D halide perovskites [8,9,10].

Excitons are important for possible applications in photonics due to the enormous oscillator strength concentrated in a narrow region of the spectrum. The optical response of excitons could be highly nonlinear, leading to the observation of four-wave mixing in halide perovskite thin films [11,12,13] and photon echo in single crystals [14,15].

The most reliable data on the exciton subsystem of bulk 3D halide perovskites can be obtained for single crystals at cryogenic temperatures using reflection spectroscopy. Low inhomogeneous broadening of the exciton resonance of single crystals and suppression of thermal broadening favours observing isolated exciton resonances and even excitonic series. Such high-quality single crystals could be grown using simple solution-processing techniques [16] or a counterdiffusion-in-gel crystallization method [17].

In this work, a study of the reflectivity from the CsPbBr${}_{3}$ single crystal in the 4–150 K temperature range is presented. Millimeter-sized CsPbBr${}_{3}$ single crystals were grown from the solution. Measurements of the reflectivity in the normal and Brewster geometry allowed us to determine its absolute value. In the high-quality single crystals, the exciton series up to n=3 is observed, as well as the effect of exciton resonance narrowing upon heating to 30 K. One of the features that was not paid attention to in previous works is a huge jump in the dielectric constant upon crossing the fundamental absorption edge. We suggest that this effect is a manifestation of the Sommerfeld enhancement, which occurs due to the correlation between the motion of electrons and holes.

## 2. Materials and Methods

### 2.1. Crystal Growth

CsPbBr${}_{3}$ halide perovskite single crystals were grown from a solution using concentrated acid: 0.6 g (2.8 mmol) of CsBr and 1.03 g (2.8 mmol) of PbBr${}_{2}$ were dissolved in 10 mL of concentrated hydrobromic acid HBr. The solution was left at room temperature for 16 h until single crystals were obtained. The resulting crystals were filtered, washed with ethanol, and stored in a dry atmosphere. The formation of the CsPbBr${}_{3}$ perovskite phase was confirmed by powder X-ray diffraction (XRD) (see [18] for XRD data and refinement).

### 2.2. Reflectivity Measurements

For reflectivity measurements, a CsPbBr${}_{3}$ single crystal was mounted in helium closed-cycle cryostat Montana Instruments and cooled down to 4 K. The halogen lamp with an yellow filter (absorption < 450 nm) was used as a light source. In normal geometry, the same 10× Mitutoyo micro objective lens was used to excite the sample and to collect the outgoing light. For Brewster geometry measurements, the light was focused by a 10 cm lens. A Glan-polarizer and an achromatic λ/2 waveplate were installed in the excitation beam. A custom-made spectrometer with a CCD camera was used to capture spectra. The photoluminescence was excited by the 450 nm CW laser.

## 3. Results

**Figure 1:**
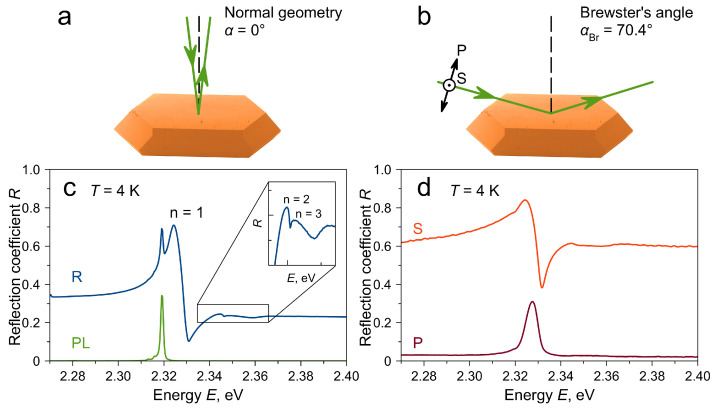
(**a**,**b**) Experimental arrangements for reflectivity measurements in normal (**a**) and Brewster (**b**) geometries. Crystal is represented by a colorized scanning electron microscope image. (**c**) Reflectivity (blue curve) and photoluminescence (green curve) spectra in normal geometry at T=4 K. Inset shows a magnified region of the reflectivity spectrum with excited excitonic states. (**d**) Reflectivity spectra in Brewster geometry for P-polarized (red curve) and S-polarized (orange curve) incident light at T=4 K.

Single crystals of CsPbBr${}_{3}$ halide perovskite were grown from a concentrated acid solution of precursor salts. This method favors the single-crystal growth of sufficiently high quality and size. Figure 1a shows a scanning electron image of the typical crystal, grown several millimeters in size. For such a dodecahedral shape of single crystals, the most developed facet corresponds to the (110) crystallographic plane [19]. Due to the size and flatness of this facet, the reflectivity spectra could be measured from it not only in normal geometry but also at grazing incidence.

To measure the reflectivity spectra near the excitonic resonance, a single crystal was mounted in a cryostat and cooled down to a temperature of T=4 K. To study reflection, two geometries were used: normal with the angle of incidence $\alpha =0^{\circ }$ (Figure 1a) and Brewster’s geometry (Figure 1b). In the latter case, when light is incident on the sample surface at the Brewster angle $\alpha =\alpha_{Br}$ with polarization in the plane of incidence (P-polarization), the reflection coefficient away from the excitonic resonance tends to zero. For the case of light incident from a vacuum, the refractive index of the material $n_{mat}$ could be determined from the value of the Brewster angle: $n_{mat}\;=\;\tan\alpha_{Br}$.

Figure 1c shows the reflectivity spectrum measured in normal geometry from the ∼20 μm area of the sample. This spectrum is dominated by a strong feature around 2.324 eV, which correlates with the reflection from a free exciton–polariton resonance with principal quantum number n=1. A typical form of such a feature is constructive interference with non-resonant reflection from the low-energy side and less pronounced destructive interference from the high-energy side. The red-shifted narrow peak at 2.319 eV is the photoluminescence (PL) signal excited by the absorbed probe light. In an isolated form, this PL signal can be obtained upon excitation of the sample with monochromatic light (Figure 1c, green curve). This PL signal is attributed to the emission of the bound exciton ∼5 meV Stokes-shifted from the free exciton resonance spectral position. Although the exact nature of the bound exciton state remains unknown, such behavior has often been observed in three-dimensional halide perovskite single crystals at low temperatures, such as MAPbI${}_{3}$ (MA${}^{+}$=CH${}_{3}$NH${}_{3}^{+}$) [15,17,20,21], MAPbBr${}_{3}$ [17] and CsPbBr${}_{3}$ [18].

On the high-energy side of the n=1 resonance, weak features can be observed, which correlates with the excited states of the exciton with n=2 and n=3. The possibility of observation of these states indicates a rather high quality of the sample. These states are part of the exciton Rydberg series and, in principle, can be used to determine the exciton binding energy accurately. However, in this case, it is desirable to observe more states, analyze the possible sources of the spectral position shift of the ground state, and build a model to accurately extract the spectral position of states from the reflectivity spectrum. States up to n=2 were reported in the reflectivity spectra from MAPbBr${}_{3}$ [6] and up to n=3 in PbI${}_{2}$ [22,23] single crystals.

Figure 1d shows the reflectivity spectrum measured in Brewster’s geometry. The Brewster angle ($\alpha_{Br}=70.4^{\circ }$) was found to be the angle at which a minimum for the high energy part of the spectrum is reached. This angle yields the background dielectric constant $n_{b}=2.81$. This value was used to normalize the reflectivity spectra. The ratio of the P- to S-polarized signal in the high-energy region was $R_{P}:R_{S}\approx 1:30$. Since the Brewster geometry is close to the grazing incidence, the signal was collected from a larger area of the sample (around 2 × 1 mm). The advantage of observing the reflection in the Brewster geometry in P-polarization is the suppression of the background non-resonant reflection from the sample surface. In this case, the reflection from the exciton resonance acquires a simple symmetric form (Figure 1d, red curve). A similar geometry is also actively used to study heterostructures based on GaAs [24,25,26,27]. The n=1 resonance has a Gaussian-like shape with full width at half maximum 5.8 meV. Excited excitonic states are smoothed by the inhomogeneous broadening due to the large area of the sample. Nevertheless, an extended feature is observed in the S-polarization spectrum up to 2.36 eV.

**Figure 2:**
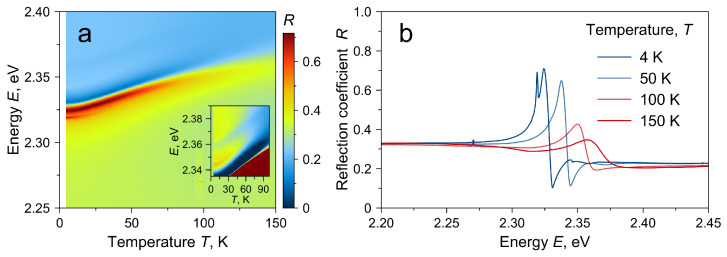
(**a**) Reflectivity spectra in normal geometry for different temperatures. The inset shows a magnified region with excited excitonic states. (**b**) Reflectivity spectra in normal geometry for different sample temperatures.

For the reflectivity spectra in normal geometry, temperature measurements were carried out in the range T=4−150 K (Figure 2a,b). The reflectivity spectra exhibit a blue shift with increasing temperature, well known for 3D halide perovskites [6,16,28]. Excited excitonic states follow the same trend but become indistinguishable at temperatures above 80 K.

To describe the spectral shape of the observed n=1 resonance, the model of uncoupled oscillators [29] was used. The real ($\varepsilon_{1}$) and complex ($\varepsilon_{2}$) parts of the dielectric constant were taken in the following form:

(1) $$\varepsilon_{1}=\varepsilon_{b}+\frac{f_{1}\left(E_{1}^{2}-E^{2}\right)}{{\left(E_{1}^{2}-E^{2}\right)}^{2}+{\left(2E\Gamma \right)}^{2}},$$

(2) $$\varepsilon_{2}=\frac{2f_{1}E\Gamma }{{\left(E_{1}^{2}-E^{2}\right)}^{2}+{\left(2E\Gamma \right)}^{2}},$$

where *E* is the light energy, $E_{1}$ is the energy of the n=1 exciton resonance, $f_{1}$ is its oscillator strength, Γ is the resonance broadening, and $\varepsilon_{b}$ is the background dielectric constant. In this case, in accordance with Fresnel formulas, the reflection coefficient *R* can be written in the following form:

(3) $$R=\frac{1+\sqrt{\varepsilon_{1}^{2}+\varepsilon_{2}^{2}}-\sqrt{2\left(\varepsilon_{1}+\sqrt{\varepsilon_{1}^{2}+\varepsilon_{2}^{2}}\right)}}{1+\sqrt{\varepsilon_{1}^{2}+\varepsilon_{2}^{2}}+\sqrt{2\left(\varepsilon_{1}+\sqrt{\varepsilon_{1}^{2}+\varepsilon_{2}^{2}}\right)}}.$$

For the low-energy limit E→0, the $\varepsilon_{2}$ vanishes and the reflection coefficient is determined by the static dielectric constant $\varepsilon_{s}=\varepsilon_{b}+\frac{f_{1}}{E_{1}^{2}}$.

**Figure 3:**
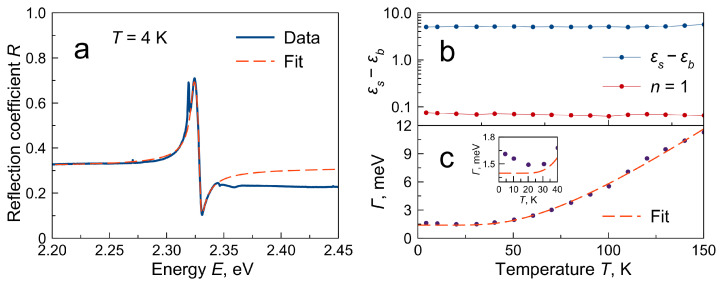
(**a**) Reflectivity spectrum in normal geometry for T=4 K and a fit by Equation (3). (**b**) Temperature dependence of the dielectric constant jump calculated as the $\varepsilon_{s}-\varepsilon_{b}$ difference (blue curve) and for the n=1 resonance as $\frac{f_{1}}{E_{1}^{2}}$ (red curve). (**c**) Temperature dependence of the broadening of the excitonic resonance extracted from fit. Dashed curve—fit by Equation (5).

Figure 3 shows the fit of the reflectivity spectrum at 4 K with the above function. The fit does not converge on the whole spectral range. Since the n=1 state is the lowest energy state in the observed spectrum, the energies above the resonance were excluded from the fit. The fit correctly describes the resonance and the low-energy part of the spectrum determined by the $\varepsilon_{s}$. However, the fitted $\varepsilon_{b}$ value is overestimated, which leads to the incorrect fit for energies above the resonance. Since the jump in the dielectric constant is proportional to the oscillator strength of the resonance, the problem could be reformulated as the underestimation of the oscillator strength in the studied region. Such an underestimation was observed earlier for other 3D halide perovskites [6,17,28], leading to the failure of providing reflectivity fit in the whole observed spectral region.

The dielectric constant jump can be determined directly from the reflectivity spectrum. If *R* is the reflection coefficient away from the exciton resonance ($\varepsilon_{2}\to 0$), then the real part of the dielectric constant can be found using the following equation:

(4) $$\varepsilon_{1}={\left(\frac{1+\sqrt{R}}{1-\sqrt{R}}\right)}^{2}.$$

Figure 3b shows the temperature dependencies of the experimental dielectric constant jump together with the jump expected from the n=1 resonance. Both values remain almost constant in the whole temperature range, which agrees with the rule of conservation of oscillator strength. However, the experimental jump value exceeds the value for n=1 by two orders of magnitude. An additional oscillator strength can be obtained by taking into account the excited states of the exciton. The oscillator strength in the excitonic series obeys the $f_{n}∼\frac{1}{n^{3}}$ law. Summing this progression for n>1 gives an increase in the dielectric constant jump by only ≈20%.

The description of the observed discrepancy could be given, taking into account both bound and unbound excitonic states. The excitonic effect plays an important role for optical properties of semiconductors near the band edge. Analytical expression for the optical absorption in the presence of Wannier excitons was given by Elliott [30]. The spectrum of the exciton is similar to the hydrogen atom and consists of a series of discrete lines and a continuum. The absorption in the continuum taking into account exciton effects exceeds the interband absorption by the so-called Sommerfeld (or Coulomb) enhancement factor. To describe the reflectivity spectra, the spectral dependencies of the real and imaginary parts of the dielectric function are needed. The analytical expression for these parameters was obtained by Tanguy [31,32]. It was shown that an enhancement is observed for $\varepsilon_{1}$ even far away from the exciton resonances due to the Kramers–Kronig relation. The Sommerfeld enhancement is proportional to the ratio between exciton binding energy and band gap energy of the material and could lead to a dramatic increase in $\varepsilon_{1}$. We suggest that the discrepancy between the jump in the real part of the dielectric constant and the extracted exciton oscillator strength observed in this and other works is a clear manifestation of this exciton enhancement.

The fit also shows the dependence of the broadening of the resonance on the temperature Γ(T). As the temperature rises to 30 K, the resonance broadening paradoxically decreases. This behavior was observed repeatedly on different samples but could be hidden in samples with larger inhomogeneous broadening [28]. Since the temperature contribution to the broadening increases with increasing temperature, this behavior can only be explained by an even faster decrease in some other broadening contribution. One of the possible mechanisms may be the thermal destruction of some centers, acting as a scattering center for excitons at low temperatures.

For higher temperatures, the exciton broadening could be described using the following dependence [33]:

(5) $$\Gamma \left(T\right)=\Gamma \left(0\right)+\frac{\Gamma_{LO}}{e^{\frac{E_{LO}}{k_{b}T}}-1},$$

where Γ(0) is the inhomogeneous broadening (broadening at 0 K), $\Gamma_{LO}$ is a parameter describing the exciton–LO–phonon interaction, and $E_{LO}$ is the optical phonon energy. Figure 3c shows the fit of the extracted data by this equation. As a result of fitting, the following values were obtained: $\Gamma_{LO}=30.3$ meV, $E_{LO}=17.8$ meV. These parameters are close to those known from the literature [28,34]. The inhomogeneous broadening in this sample was found to be Γ(0)=1.4 meV, which is an order of magnitude less than that known from the literature for other CsPbBr${}_{3}$ samples [28]. This indicates the high quality of the grown single crystals and unlocks the study of fine optical effects.

## 4. Discussion

In this work, the reflection from CsPbBr${}_{3}$ halide perovskite single crystals at cryogenic temperatures was studied. The high quality of the crystal made it possible to observe the excited states of Wannier–Mott excitons up to n=3 and the effect of a decrease in the broadening of the n=1 exciton resonance in the initial region of heating from 4 K to 30 K. Three-dimensional halide perovskites have a huge oscillator strength of exciton resonances, which manifests itself with the reflection coefficient tending to unity even in the presence of substantial broadening. In the studied CsPbBr${}_{3}$ crystal the reflection coefficient up to 70% was observed at the lowest temperatures.

Another observation known from the literature is the discrepancy between the jump in the dielectric constant near the material band edge and the oscillator strengths of individual exciton resonances. Although the exact value of the dielectric constants of this material at low temperatures requires further clarification and independent verification, the qualitative scale of this discrepancy is significant. In this work, this discrepancy is explained by the need to take into account the Sommerfeld excitonic enhancement factor. A complete theory that takes into account this enhancement, the exciton series, and polariton effects must be developed in the future to accurately describe the reflection spectra of these unique perovskite materials.

## Data Availability

The data presented in this study are available on request from the corresponding author.

## Abbreviations

The following abbreviations are used in this manuscript:

PL	Photoluminescence
